# Effectiveness and Efficacy of Thermoformed and 3D Printed Aligners in Correcting Malocclusion (Spacing) and Its Impact on Periodontal Oral Health and Oral Microbiome: A Double-Blinded Parallel Randomized Controlled Multicenter Clinical Trial

**DOI:** 10.3390/microorganisms10071452

**Published:** 2022-07-19

**Authors:** Shahnawaz Khijmatgar, Margherita Tumedei, Massimo Del Fabbro, Gianluca Martino Tartaglia

**Affiliations:** 1 Department of Biomedical, Surgical and Dental Sciences, Università degli Studi di Milano, 20122 Milan, Italy; margherita.tumedei@unimi.it (M.T.); massimo.delfabbro@unimi.it (M.D.F.); gianluca.tartaglia@unimi.it (G.M.T.); 2 IRCCS Fondazione Ca’Granda IRCCS Ospedale Maggiore Policlinico, 20122 Milan, Italy

**Keywords:** thermoformed, aligners, 3D, spacing, oral microbiome, oralhealth, RCT, randomised

## Abstract

Aligners are the common devices used in orthodontics for the correction of malocclusion. Various materials and techniques are employed to fabricate aligners. One of those includes thermoformed and 3D aligners. These aligners can be worn for several days, and their impact on periodontal health is not known. Therefore, the aim of our protocol is to determine the effectiveness of these aligners in correcting malocclusion and their impact on periodontal health and oral microbiome. A double-blinded randomized controlled clinical trial with a total of *n* = 60 patients will be included with *n* = 30 in each group (Test: 3D printed aligners and Control: Thermoformed). The evaluation of oral health indices such as basic periodontal examination (BPE), periodontal screening and recording (PSR) that provide the status of periodontal health will be recorded. The oral microbiome assessment will be conducted with polymerase chain reaction (PCR). The primary endpoint will be the correction of malocclusion, and the secondary end point will be the status of periodontal health and oral microbiome. The duration of follow-up for each group will be 7 days for periodontal health and oral microbiome and 6 months for the space closure of 5 mm by 3D and thermoformed aligners.

## 1. Introduction

The prevalence of malocclusion varies among different regions and races of the population. In the United States, the frequency of malocclusion is determined by Nutrition Examination Survey (NHANES III) and the National Health. It emerged that the percentage of adults having misaligned incisors was 65%, and the percentage of adults having crowding severe enough to interfere with function and social interactions was 15% [1]. Treatment of malocclusion has become more common among the population of United States and other developing countries. There is a sense of security that the correction of maligned teeth improves the quality of life (QoL) significantly [1]. Generally, the patients believe that it provides aesthetic, psychosocial and functional benefits. There is even an economic burden for this orthodontic problem, which affects patient’s parents and families. In addition to the treatment costs, other factors such as school absence, work and productivity loss should also be considered. Interceptive therapy for malocclusion treatment is less costly and less complicated than the traditional approach but, in some situations, do not result in comparable dental outcomes. O’Brien and colleagues (2009) have looked at the cost differences between one- and two-phase treatments [2]. The authors found that the cost will be significantly higher in two-phase treatments as compared to one-phase treatments. Deans and colleagues (2009) demonstrated the cost-effectiveness of orthodontic treatment in Europe [3] and has a significant impact on orthodontic treatment on cost. In addition to the cost, conventional orthodontic treatment for correcting malocclusions (removable and fixed) has a greater tendency to cause periodontal problems. These include gingivitis, periodontitis, gingival recession, hypertrophy, fenestrations, dehiscence, dark triangles, and interdental folds. Additionally, the formation of biofilm is the most important indicator for the initiation and progression of periodontal disease during orthodontic treatment.

Traditionally, malocclusions are corrected with removable and fixed prosthesis appliances [4]. They bring both dental and skeletal changes resulting in the correction of malocclusions, but these have both advantages and disadvantages. They are visible, metallic, long time wearing, and lack compliance of the patients. Recently, clear aligners have been introduced for correcting intrusion, extrusion, spacing, buccolingual angulation, anterior buccal and palatal angulation, etc. [5,6,7,8]. There are different commercially thermoplastic materials to produce the clear aligners on dental models that express the tooth movement [9,10,11,12]. Both conventional fixed orthodontic treatment and aligners correct malocclusions significantly, but aligners have the advantage of correcting malocclusions by the segmented movement of the teeth and shortened duration of treatment. However, aligners have drawbacks, such as achieving good occlusal contacts, retention, and tooth torque. The evidence on the effectiveness of 3D aligners in correcting malocclusion in comparison to conventional thermoformed aligners is lacking, and a more recent update on the systematic reviews and meta-analysis in this issue is warranted [13,14,15,16,17,18]. Both therapies also have an impact on the oral microbiota and change in oral flora over a period of time. Before any 3D direct-printed aligners are adopted, 3D aligners need to be compared with traditional thermoformed aligners. To date, the dimensional accuracy of direct-printed aligners has not been tested against those of traditional thermoformed aligners. Dimensional accuracy is a common research topic in dentistry to assess dimensional precision.

A recent randomized clinical trial confirmed that treatment duration and chair time in mild-to-moderate cases appear to be significantly short over conventional systems [19]. There is also a lack of evidence on the use of aligners in space closure and its impact on periodontal health [20]. There is a need for evidence in conducting well-designed clinical trials.

The aim of the present study is to determine the effectiveness and efficacy of 3D aligners in correcting malocclusions as compared to that of thermoformed method and to determine the periodontal health status during and after 3D aligners and thermoformed aligners therapy.

### 1.1. Aim 1: Effectiveness and Efficacy of Clear Aligners (Invisalign) in Correcting Malocclusions

The clear aligners were introduced in the 1940s, but due to lack of evidence and promotion, it was not widely practiced and adopted. Later, due to the development of biomaterials in dentistry and 3D technology, it became very much popular. Recently, there have been more expert opinions in favor of clear aligners, and there is a lack of evidence in correcting a wide range of malocclusions [21]. Not many studies described the effectiveness of treatment with 3D and thermoformed clear aligners and its impact on periodontal health, generating doubts among clinicians on the efficacy of the device.

Therefore, a well-designed parallel randomized controlled clinical trial will help to overcome the problem. The spacing >5 mm correcting it by using 3D clear aligners and compared with thermoformed aligners therapy.

### 1.2. Aim 2: Periodontal Health Status and Oral Microbiome

The second aim is to determine the periodontal health status during and after 3D clear aligners and thermoformed aligner therapy.

Clear aligner treatment (CAT) has been described as a secure and confident orthodontic procedure for patients. Recently, adult patients have sought a more aesthetic and comfortable orthodontic treatment than fixed appliances. To meet these needs, the treatment with transparent aligners (CAT) was introduced. The clear aligners cover the keratinized gingiva for a long period of time, and it is very important from a clinical point of view that CAT does not cause negative effects on periodontal tissues. Both conventional treatment and aligners cause a shift in the increase in anaerobes and periodontal pathogens and a decrease in commensal bacteria in the supra-gingival microbiome, but there are different types of aligners, i.e., 3D aligners and thermoformed aligners that can cause a periodontal reaction due to several factors such as the presence of systemic conditions, host resistance, and the amount and composition of dental plaque. Smoking and the patient’s lifestyle can compromise periodontal health and support. Oral hygiene procedures play a primary role in periodontal health during orthodontic treatment. Therefore, the objective is to determine the status of periodontal status and oral microbiome in 3D and thermoformed CAT.

## 2. Study Design

### 2.1. Preliminary Studies

In relation to the effectiveness and efficacy of clear aligners, there are two Randomized Clinical Trials (RCT), five prospective non-randomized and four retrospective non-randomized trials. There was moderate level of bias in these studies. The Invisalign clear aligners are good in managing spacing, anterior crowding and in adjusting marginal aligners that compare the outcome with respective of periodontal conditions and oral microbiome. We expanded this hypothesis generating the findings to design, set up and recruit patients into our randomized controlled clinical trial to prospectively test that the clear aligners are good in managing spacing and crowding and can bring down the mortality of root resorption that occurs due to traditional methods. Additionally, it helps to determine its impact on periodontal oral health by assessing the saliva and biofilm under ELISA and PCR methods.

### 2.2. Trial Design

**Figure 1:**
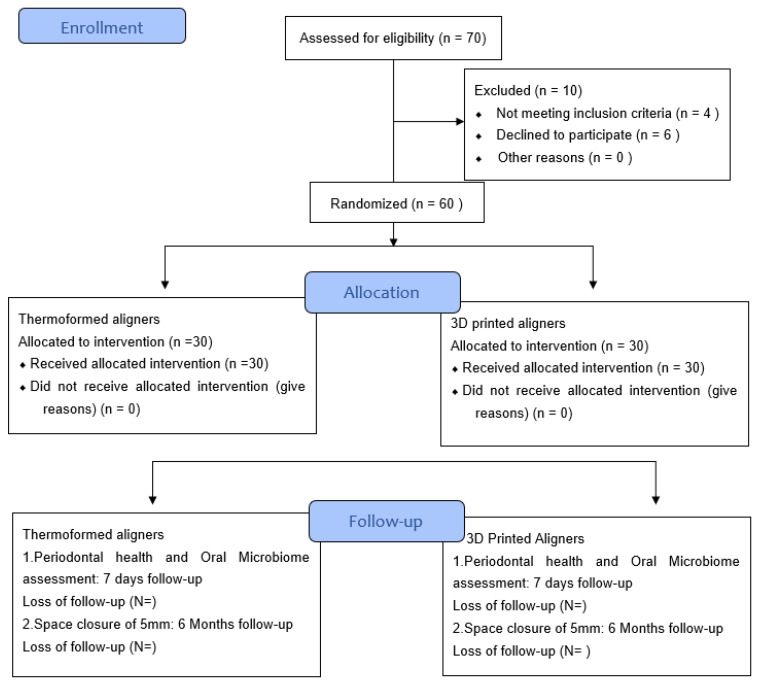
CONSORT 2010 Flow Diagram.

We propose to conduct a double-blinded randomized controlled clinical trial in patients who have >5 mm of spacing in both maxilla and mandible and cases with skeletal class I coming to the department of Orthodontics and Dentofacial Orthopaedics at Fondazione IRCCS Cà Granda Ospedale Maggiore Policlinico di Milano, SST clinics and Università degli Studi di Bari and Naples Clinica Odontoiatrica. Patients in both arms will receive adhered support and will be monitored more frequently than currently recommended by WHO guidelines. The first arm will undergo a traditional method of managing of closure of spacing by thermoformed aligners, and the second aim will be treated with thermoformed and 3D printed aligners. Eligible patients are randomized to the control arm or the intervention arm with a 1:1 ratio using a blocked randomization scheme. The patients will be followed for 6 months (Figure 1).

There will be an initial assessment with detailed case history, extra-oral and intra-oral examination, and clinical radiographs. A diagnosis and treatment plan will be framed. Pre- and post-operative samples of biofilm and saliva will be collected to determine the inflammatory and immunological response. The bridging and early colonizers such as *Fusobacterium nucleatum* (*F. nucleatum)*, pathogenic species such as *Porphyromonas gingivalis (P. gingivalis)*, microbial communities and the composition of the biofilm will be determined by ELISA and PCR. This will help to answer the questions such as if any infection is present, which bacteria colonize, whether we should use antibiotics, etc. In addition, 16 s-ribosomal RNA signatures for identification of the microbial composition of biofilm structures.

***Baseline information:*** (1) A Patient Measured Outcome Questionnaire (PROM) was completed to determine the impact of oral health on quality of life. The PROMs are dozens of tools that allow you to measure the important results for the patient, for example, cost, symptom relief, quality of care, improved oral health, and overall health. It is evaluated after each intervention. (2) Numerical rating scale (NRS) for measuring the pain of patients before and after the intervention. (3) Periodontal health status will be assessed by basic periodontal examination (BPE), Periodontal screening and recording index (PSR index), Basic Periodontal Examination (BPE) scores, Oral Hygiene Index simplified (OHIS), Saliva (Protease activity in saliva) to determine the immune-inflammatory processes by ELISA and biofilm by polymerase chain reaction (PCR). ***Inclusion criteria:*** The eligible cases will be identified during routine clinical visits. CAT is indicated in cases of slight crowding (1–5 mm), spacing problems (1–5 mm), deep overbites (Class II, div. 2), narrow arches requiring expansion, absolute intrusion (one or two teeth), severe crowding with the extraction of the lower incisor and molars requiring distal tipping. Contraindications are crowding and spacing problems greater than 5 mm, anterior-posterior skeletal problems greater than 2 mm, relationship discrepancies and centric occlusion, severely rotated and inclined teeth, open bite, dental extrusion cases, cases with more missing teeth and teeth with short clinical crowns.

Written informed consent will be obtained before the inclusion of the study. An entry form will be completed to determine the eligibility and to collect the baseline characteristics such as periapical radiographs, panoramic radiographs, models, casts, cephalometric tracings, CBCT scans, etc. Patients will be randomized in thermoformed and 3D clear aligners. All non-extraction cases except third molar extraction will be included. ***Exclusion criteria:*** Patients with deciduous and mixed dentition will not be included, and patients with systemic disease, developmental abnormalities, pregnant patients, mentally retarded patients and immunocompromised patients will not be included in the study.

### 2.3. Primary End Point

1. Five-millimeter space closure: To determine the successful outcome of the 3D and thermoformed aligners, a 5 mm of space closure is considered a successful outcome.
2. Debris Index: (The buccal scores) + (The lingual scores)/(Total number of examined buccal and lingual surfaces).
3. Calculus Index: Calculus Index = (The buccal scores) + (The lingual scores)/(Total number of examined buccal and lingual surfaces).
4. Oral Hygiene Index simplified (OHIS): OHIS estimates the extent and amount of debris and calculus present on the tooth in each quadrant of the mouth. It demonstrates the oral hygiene status of the mouth. It is estimated using the debris index and calculus index provided by Greene and vermillion (1964).
5. Plaque Index: The plaque index estimates the amount of plaque on the tooth surface in each quadrant. 0—Absence of plaque, stain or debris; 1—plaque covering no more than 1/3rd of tooth surface; 2—subtle plaque more than 1/3rd but not more than 2/3rd; 3. Plaque cover more than 2/3rd of the tooth surface.
6. Bleeding Index: A modified Sulcus Bleeding Index (mSBI) will be used to record the bleeding from the sulcus and to determine the inflammation, as it is the early indicator of inflammation (clinically).
7. Basic periodontal examination index (BPE): Periodontal status will be determined using the BPE scoring.
8. Root Resorption: Basic periodontal examination such as hard tissues will be conducted using a combination of bitewing and peri-apical radiographs following selection criteria for dental radiography guidelines.
9. Biofilm/Saliva: Saliva (Protease activity in saliva) to determine the immune-inflammatory processes by ELISA and biofilm (protease activity) by polymerase chain reaction (PCR).
10. Oral microbiome: A standard estimation of the oral microbiome will be carried out using 16S ribosomal RNA gene sequencing analysis.

### 2.4. Secondary End Point

White Spot Lesions, Patient Reported Outcome Measures (PROM’s).

## 3. Statistical Analysis

The sample size was calculated using a two-sided test by comparing two independent means of control and test between groups with a statistical power of 0.80, alpha of 0.05, with an allocation ratio of 1:1 using STATA 17.0 statistical software. The mean of space closure of 3 mm in control and 5 mm in the test group with sd for control 2.5 and test 2.0 demonstrated that a sample of a least 22 subjects for both groups must be recruited (a total of 44 patients should be recruited). In order to compensate for the loss of follow-up, an increase in patient number *n* = 70 will be recruited.

A descriptive statistic will be carried out for all the indices before and after intervention with 3D and thermoformed aligners. If the data are normally distributed, a parametric test will be carried out. The non-parametric test will be used for the analysis of data that are not normally distributed. A regression analysis to determine the relationship between the dependent and independent variables and for modeling the future relationship between them.

***Safety measurements and adverse events:*** All patients will be monitored for general health and for serious adverse events related to the drug intervention. General and oral assessment including weight, blood pressure, pulse rate, skin, lymph node size, and thyroid palpation will be recorded. There will be no compensation for any adverse events or withdrawal from the study.

***Challenges and Limitations: Follow-up:*** There are chances that there will be a loss of compliance from the patient. To overcome this problem, the patient will be educated and will increase awareness in regard to the type of treatment receiving and its impact on aesthetics.

## Data Availability

None data collected as this is a protocol.
